# Role of Matrix Metalloproteinases in Angiogenesis and Its Implications in Asthma

**DOI:** 10.1155/2021/6645072

**Published:** 2021-02-13

**Authors:** Khuloud Bajbouj, Rakhee K. Ramakrishnan, Qutayba Hamid

**Affiliations:** ^1^College of Medicine, University of Sharjah, Sharjah, UAE; ^2^Sharjah Institute for Medical Research, University of Sharjah, Sharjah, UAE; ^3^Meakins-Christie Laboratories, McGill University, Montreal, QC, Canada

## Abstract

Asthma is a chronic airway disorder associated with aberrant inflammatory and remodeling responses. Angiogenesis and associated vascular remodeling are one of the pathological hallmarks of asthma. The mechanisms underlying angiogenesis in asthmatic airways and its clinical relevance represent a relatively nascent field in asthma when compared to other airway remodeling features. Matrix metalloproteinases (MMPs) are proteases that play an important role in both physiological and pathological conditions. In addition to facilitating extracellular matrix turnover, these proteolytic enzymes cleave bioactive molecules, thereby regulating cell signaling. MMPs have been implicated in the pathogenesis of asthma by interacting with both the airway inflammatory cells and the resident structural cells. MMPs also cover a broad range of angiogenic functions, from the degradation of the vascular basement membrane and extracellular matrix remodeling to the release of a variety of angiogenic mediators and growth factors. This review focuses on the contribution of MMPs and the regulatory role exerted by them in angiogenesis and vascular remodeling in asthma as well as addresses their potential as therapeutic targets in ameliorating angiogenesis in asthma.

## 1. Introduction

Asthma is a highly heterogeneous chronic respiratory disease characterized by inflammation, hyperresponsiveness, and remodeling of the airways. Frequent asthma exacerbations triggered mainly by allergen exposure or viral or bacterial infections are primarily caused by chronic inflammatory processes that progress to a series of structural changes to the bronchial wall, including the resident airway epithelium, basal membrane, fibroblasts, smooth muscles, and blood vessels.

Angiogenesis is characterized by the emergence of new blood vessels from preexisting endothelial lined vessels. It is a normal physiological process that plays an important role in development and wound healing. At the same time, it is also a fundamental process in the pathogenesis of various diseases, such as cancer, obesity, rheumatoid arthritis, psoriasis, cardiovascular diseases, and asthma. Proteolysis being a key regulator of angiogenesis, proteases such as matrix metalloproteases (MMPs), the closely related family of a disintegrin and metalloprotease (ADAM) domain proteins, which includes ADAM and ADAMTS (a disintegrin and metalloprotease domain with thrombospondin motifs), as well as cysteine and serine proteases, have been implicated in regulating the angiogenic process.

The various proteolytic enzymes within the MMP family share a similarity in their structures and collectively are capable of breaking down the various known extracellular matrix (ECM) proteins. The growing family of MMPs comprise of members including collagenases, gelatinases, stromelysins, membrane-type MMPs (MT-MMPs), matrilysins, and various other MMPs. Depending on the presence of a transmembrane domain, the MMPs are broadly classified into two—MT-MMPs and secreted MMPs. As part of the homeostatic mechanism, the activated MMPs are deterred by a group of endogenous inhibitors called tissue inhibitors of metalloproteases (TIMPs). Four members of this family, namely, TIMP-1, TIMP-2, TIMP-3, and TIMP-4, have been identified till date.

This review discusses the current understanding of MMPs and their role in the development of angiogenesis in asthma. Furthermore, we summarize the therapeutic modalities currently under investigation to target MMPs and their implications in improving angiogenesis and vascular remodeling in asthma.

## 2. Tissue Remodeling in Asthma

Tissue remodeling refers to modifications associated with the normal composition and structural organization of tissues. This can occur in a wide range of tissues and organs, including the airways, lung [1], blood vessels [2], heart [3], and gastrointestinal tract [4, 5]. Airway remodeling is a characteristic feature in patients with pulmonary disorders, such as asthma, chronic obstructive pulmonary disease (COPD), and cystic fibrosis. This event is mainly driven by inflammatory mediators that bring about cellular and structural changes resulting in thickening of the airway wall, thereby leading to airway narrowing and airflow limitation. Airway remodeling in asthmatic patients involves a wide array of pathophysiologic features, including epithelial changes, subepithelial fibrosis, increased smooth muscle mass, and vascular changes, primarily around the large airways. These structural modifications thus affect all cellular layers of the bronchial wall, from airway epithelium, basement membrane, subepithelial fibroblasts, smooth muscles, and cartilage to blood vessels lining the airway wall. Whereas remodeling in patients with COPD involves structural changes mainly to the small airways, remodeling in patients with cystic fibrosis is characterized by fibrotic, glandular, muscular, and vascular changes throughout the lung.

Structural changes in the airway walls are predominantly detected in the more severe forms of asthma, where they are characterized by ECM remodeling, epithelial desquamation, goblet cell hyperplasia, prominent smooth muscle area, vascular remodeling, collagen deposition below the basement membrane, loss of cartilage integrity, and elastolysis [1]. The clinical consequences of remodeling are severe thickening of the airway walls leading to bronchial obstruction during an asthma attack [6]. Some of the earliest structural remodeling changes are observed in the basement membrane, where excessive ECM deposition leads to its thickening and reduction in elasticity. Airway smooth muscle (ASM) cell hyperplasia and hypertrophy are also demonstrated within the smooth muscle layer. Additionally, the mucosal glands are enlarged and associated with excessive mucus production. The continuing inflammation is further linked with the persistence of exacerbations and nonspecific airway hyperresponsiveness (AHR). Besides, the degradation of elastin and cartilage may result in decreased airway wall stiffness and increased airway narrowing. In asthma, remodeling is usually detected in biopsy specimens but is not always clinically demonstrated [7]. Nevertheless, structural remodeling of the bronchial tree paves way for increased AHR and progressively more severity in the course of the disease.

## 3. Angiogenesis in Asthma

The airways are supplied with blood vessels localized in the bronchial smooth muscle layer, as well as through the capillary network in lamina propria [8]. The bronchial blood vessels are known to undergo alterations in their density, dilation, and permeability under both physiological and pathological conditions. As early as 1960, increased vascularity has been reported in the bronchial mucosa in association with the pathology of asthma [9]. Since the mechanisms underlying angiogenesis in asthmatic airways and its clinical relevance represent a relatively nascent field in asthma when compared to other airway remodeling features, there is a growing interest among scientists in the fields of angiogenesis and neovascularization in asthmatic patients.

Angiogenesis is a process of new blood vessel formation, and it involves several stages that are highly regulated. The early stage of angiogenesis occurs in a preexisting blood vessel localized in close proximity to the inflammatory process. This stage involves increased blood vessel permeability, endothelial cell activation by growth factors, and increased endothelial mitosis. The ensuing second stage involves the degradation of the endothelial basement membrane by matrix metalloproteinases. This is followed by the migration of endothelial cells towards the different angiogenic factors and the establishment of branch points and capillary lumen. The final stage comprises of modeling and stabilization of the new capillary vessel. In the last leg, the endothelial cell junctions are tightened, the basement membrane is established, and the pericytes are recruited.

Several studies have suggested an abnormal increase in the number and size of microvessels within bronchial tissue in remodeled asthmatic airways [10–12]. These vascular changes were observed below the basal lamina in the space between the muscle layer and the surrounding parenchyma. The vascular bed in bronchial lamina propria of asthmatic subjects was significantly enriched with blood vessels than in nonasthmatic subjects [13]. The vasculature also showed marked structural alterations in terms of edematous walls and subendothelial basement membrane thickening. Besides intense eosinophil recruitment and intravascular activation, the intra-arteriolar muscular formations in asthmatics exhibited hypotrophic or atrophic myocytes and fibrosis. Bronchial mucosa microvascularization, in addition to being increased in asthmatic patients in comparison to control, was also found to correlate with the clinical stage of the disease and forced expiratory volume in one second (FEV1) values [13, 14]. Notably, severe asthmatic patients demonstrated a 46% increase in capillary vasculature in the bronchial submucosa when compared to controls [14].

Furthermore, the infiltrating eosinophils, basophils, and mast cells as well as the resident epithelial, endothelial, and ASM cells secrete various angiogenic factors, including hypoxia inducible factor (HIF), vascular endothelial growth factor (VEGF), and angiopoietins, which direct the development of angiogenesis in the submucosa. Airway obstruction and chronic inflammatory processes in asthmatic airways are known to cause the induction of a locally restricted hypoxic environment which further triggers the initiation of angiogenesis [15]. Elevated HIF levels have been reported across both endobronchial biopsies and in the bronchoalveolar lavage (BAL) fluid of asthmatics [16, 17]. The increased HIF subunits (HIF-1*α* and HIF-1*β*) in lung tissues also further correlated with VEGF levels [17]. VEGF is known to inhibit the apoptosis of vascular endothelial cells. Elevated VEGF and cysteinyl leukotrienes (Cyst-LTs) levels were detected in asthmatic sputum supernatant when compared to normal subjects [18]. In this study, the authors suggest that Cyst-LTs modulate vascular permeability by stimulating VEGF expression. VEGF causes vessel dilation and edema by increasing the permeability of these abnormal blood vessels [19] resulting in airway thickening and subsequent narrowing. Thus, these vessels, in addition to providing nutrition to the airways, are the source of inflammatory cells and plasma-derived mediators and cytokines [10]. The imbalance in the levels of VEGF and angiopoietin-1 contributes to these vascular abnormalities in asthmatic airways [20]. Angiopoietins play a particularly important role in the final stage of angiogenesis where they stimulate the migration of pericytes and help stabilize the newly formed capillary tubes.

## 4. Matrix Metalloproteinases in Asthma

In the airways, the basement membrane supporting the surface epithelium is composed of several layers: the basal lamina and the lamina reticularis. In asthma, the basal lamina is of normal thickness, whereas the reticular layer is thickened leading to subepithelial fibrosis of the airways. Clinically, the thickening of the lamina reticularis is a characteristic early feature of the asthmatic bronchus. These features represent one of the most common remodeling patterns of asthma. The ECM produced by connective tissue cells forms a complex network filling the extracellular space of the submucosa. In addition to their role in supporting and maintaining the tissue structure, ECM influences many cellular functions such as development, migration, and proliferation [21]. Abnormal deposition of ECM elements has been described in the submucosal and adventitial areas of the large and small airways of asthmatic patients [22–25]. Although deposition of collagen IV and elastin appears to be decreased in the airway walls of asthmatic patients, collagens I, III, and V, fibronectin, tenascin, hyaluronan, versican, and laminin are increased compared with those seen in healthy subjects [26–30].

MMPs belong to a family of zinc-dependent endopeptidases that play key roles in both physiological processes, such as wound healing [31, 32], as well as in pathological conditions, including inflammation [33] and fibrosis [34]. MMPs are well known to degrade ECM and to regulate cell signaling through the cleavage and processing of bioactive molecules, including growth factors and cytokines. Multiple cell types secrete MMPs including both inflammatory cells, such as macrophages [35] and leukocytes [36, 37], and airway structural cells, such as airway epithelial cells [38, 39], fibroblasts [40, 41], and smooth muscle cells [42]. Several subclasses of MMPs have been identified, including collagenases, gelatinases, stromelysins, and membrane-type MMPs that can degrade many ECM proteins including collagens, fibronectin, laminin, proteoglycans, entactins, and elastin. Normally, MMPs are secreted as inactive proenzymes, which are activated by the loss of the propeptide under physiologic conditions [21]. MMP expression and activity are tightly regulated by the action of endogenous inhibitors of MMPs, referred to as tissue inhibitors of metalloproteinases (TIMPs). Excessive ECM breakdown resulting from an MMP-TIMP imbalance occurs in various pathologic processes, including inflammation, chronic degenerative diseases, and tumor invasion.

The restoration of functional connective tissue is a major goal of the wound healing process. This regenerative event requires the deposition and accumulation of collagenous and noncollagenous ECM molecules as well as the remodeling of ECM by MMPs. The inhibitors, TIMP-1 and TIMP-2, obstruct the activities of all known MMPs and as such play a key role in maintaining the balance between ECM deposition and degradation in different physiologic processes. Loss of balance in the expression of proteinases and inhibitors may result in tissue degradation in inflammatory diseases [43].

MMP-9 was among the first to be implicated in asthma pathogenesis, where abundant *MMP-9* mRNA expression was noted in submucosal regions of asthmatic bronchial biopsies when compared to normal subjects, especially within the eosinophils in asthmatic tissues [44]. Interestingly, the protein expression was not abundantly seen in the inflammatory cells, but immunoreactivity was rather detected in the ECM. Additionally, neutrophils are another important source of MMP-9 in allergic asthmatic patients [36]. In a 5-year follow-up study, increased MMP-9 and MMP-9/TIMP-1 ratio in the fast FEV1 decline group in asthmatic bronchial biopsy specimens and alveolar macrophages imply their contribution to a greater decline in lung function of patients with chronic asthma [45].

## 5. Immunomodulatory Role of MMPs in Asthma

MMPs play a key role in immune cell development, effector function, migration, and ligand-receptor interactions. They carry out ectodomain shedding of cytokines and their cognate receptors. MMPs influence immune responses by regulating signal transduction pathways downstream of tumor necrosis factor receptor, interleukin- (IL-) 6 receptor, epidermal growth factor receptor, and Notch signaling, which are all pertinent for inflammatory responses [43].

Inflammation, a key hallmark feature of asthma, is also regulated by MMPs, which exhibit both proinflammatory and anti-inflammatory properties. MMPs facilitate both the recruitment and clearance of inflammatory cells through the cleavage of inflammatory mediators such as chemokine substrates [46]. For instance, MMP-7 (matrilysin)-mediated shedding of syndecan-1, a heparan sulfate proteoglycan, is required for establishing a chemokine gradient for the transepithelial migration of leukocytes into the alveolar air spaces [47].

IL-13, a T helper type 2 cytokine, demonstrated the ability to regulate most of pathological processes in allergic asthma. For example, mice with inducible lung-targeted overexpression of IL-13 showed the pathogenic effects of IL-13 on inflammation and airway remodeling [48, 49]. IL-13 overexpression was found to be sufficient enough to induce most of the features of allergic asthma seen in human patients in other murine models of allergen challenge. These IL-13 transgenic mice harbored significantly high levels of *MMP-2*, *MMP-9*, *MMP-12*, *MMP-13*, *MMP-14*, and *TIMP-1* mRNA expression and MMP-2, MMP-9, and MMP-12 activity in the lung tissue as compared to nontransgenic animals [49]. This highlights the pathological and immunomodulatory role of MMPs in allergic asthma.

MMP-deficient mouse models have revealed important information regarding their role in airway inflammation in asthma. MMPs, in particular MMP-9, are secreted by inflammatory cells following allergen provocation and in response to T helper type 2 cytokine signaling [50]. These factors facilitate inflammatory cell egress from the tissues to the airway lumen. Additionally, inflammatory cell- and structural cell-derived MMPs also contribute to AHR and remodeling by altering ECM turnover, which affects smooth muscle contraction, airway fibroblast invasion, and submucosal accumulation of collagen. Furthermore, MMP-induced regulation of cell signaling through proteolytic shedding and activation of key growth factors, such as TGF-*β*1, stimulates airway cell proliferation and modulates matrix production, contributing to airway fibrosis [50]. MMP-9 and MMP-2 have been implicated in the infiltration of eosinophils through the basement membrane into the asthmatic airway walls and the subsequent induction of AHR [51, 52]. This immunomodulatory role of MMPs in asthma provides the attractive possibility of MMP inhibition as a therapeutic option in bronchial asthma.

## 6. Role of MMPs in Angiogenesis and Asthma

Angiogenesis involves the destruction of the vascular basement membrane and remodeling of the ECM, which paves way for the migration and proliferation of endothelial cells as well as the synthesis of new matrix components. MMPs play an important role in this disruption and neovascularization process, thus constituting a key element in the pathophysiological mechanisms underlying vascular remodeling in asthma. Airway inflammation entails the migration of activated inflammatory cells from the circulation into the airway wall towards the site of injury, and the airway structural cells closely interact with the ECM components in promoting angiogenesis in asthmatic airways. MMPs have largely been studied in aiding this extravasation across the vascular and airway membranes.  Table 1 enlists the various MMPs (in their order of relevance) implicated in promoting angiogenesis in asthma.

The major role of MMPs is the breakdown of the vascular basement membrane and ECM paving the way to tissue remodeling and angiogenesis. MMPs cover a broad range of angiogenic functions, from the degradation of the preexisting basement membrane and ECM to the release of a variety of angiogenic and growth factors as well as stimulation of endogenous angiogenic inhibitors. MMPs thus contribute to vascular remodeling through multiple mechanisms involving proteolysis of type I collagen, regulation of perivascular or smooth muscle cells, modification of platelet-derived growth factor (PDGF) signaling, and processing and mobilization of VEGF [53]. As discussed earlier, among the numerous MMPs, MMP-9 is the most commonly implicated in asthmatic airways. MMP-9 is well known to trigger the angiogenic switch in carcinogenesis [54]. The elevated levels of MMP-9 in asthmatic airways make MMP-9 a likely pathological angiogenic player in asthma as well. The airway infiltrating cells, including mast cells and basophils, are sources of VEGF in the airways [55, 56]. In a study by Lee et al., VEGF signaling was found to regulate MMP-9 expression in a murine model of asthma with the inhibition of VEGF receptor contributing to the downregulation of MMP-9 [57]. Furthermore, VEGF receptor inhibition also led to a reduction in plasma extravasation as well as the number of inflammatory cells (eosinophils, lymphocytes, and neutrophils) in BAL fluids, suggesting a role for MMP-9 in promoting the migration of inflammatory cells across the endothelial basement membrane. *Mmp-9*, *Mmp-2*, and *Mmp-14* mutant mice show defects in angiogenesis despite normality in their VEGF and VEGFR2 levels [58–60]. The reduction in bronchial vascular extracellular remodeling brought about by inducible NO synthase (iNOS) inhibition was found to be associated with MMP-9/TIMP-1 vascular expression [61], reinforcing the regulatory potential of MMP expression on vascular remodeling.

Extracellular vesicles (EVs) containing MMPs are novel mediators of ECM remodeling [62]. Several cells of the respiratory system, including bronchial epithelial cells, vascular endothelial cells, alveolar macrophages, eosinophils, neutrophils, and fibroblasts, secrete EVs that are crucial for intercellular communications [63, 64]. Exposure to tobacco smoke reportedly induced the release of proteolytic EVs from human macrophages [65]. The gelatinolytic and collagenolytic activities exhibited by these EVs can be predominantly attributed to MT1-MMP/MMP-14. MMP-containing EVs have been largely studied in cancer models, where they were shown to promote angiogenic activities *in vitro* as well as *in vivo* [66, 67]. Platelet-derived microvesicles stimulated the expression of angiogenic factors, MMP-9, VEGF, and IL-8, which promoted angiogenesis in a human syngeneic mouse model of lung cancer [68]. Since asthma patients demonstrate increased levels of EVs [69, 70], it is highly likely that MMP-containing EVs contribute to angiogenesis in asthma.

## 7. MMP-Targeted Therapeutic Advances in Asthma

Considering the pathological role of angiogenesis in asthma and the limited effectiveness of standard asthma therapy in ameliorating airway remodeling in asthma underlines the importance of identifying potential targets to control the development of angiogenesis and associated vascular remodeling in asthma. The identification of MMPs as crucial regulators of the angiogenic process had led to the development of therapeutic strategies targeting MMPs. Some of the therapeutic strategies targeting MMPs that are currently under investigation include the use of small molecular MMP inhibitors, TIMPs, antisense technologies, and blocking antibodies, as have been extensively reviewed in [88–90].

Although the airways are equipped with physiological or endogenous inhibitors such as TIMPs, diseased airways cripple the MMP/TIMP ratio favoring pathogenesis and airway remodeling. TIMP-1 antagonizes MMP-9 activity, and a lower MMP-9/TIMP-1 ratio in sputum from untreated stable asthmatics suggests an overproduction of TIMP-1 over MMP-9 in patients with stable asthma [91]. Since TIMP-1 shows anti-angiogenic activity by blocking the endothelial cell response to angiogenic factors and cell migration [92–96], this natural endogenous activity of TIMP-1 may be harnessed to impede angiogenic activity in asthma. However, altering the delicate protease/anti-protease balance needs to be considered with caution as this strategy may backfire leading to worsening outcomes in asthma. The different binding rates and binding affinity of TIMPs constitute another challenge in using it to target the different MMPs.

There are also several exogenous MMP inhibitors available such as hydroxamate derivatives including batimastat, marimastat, and ilomastat. Although these agents have shown effectiveness in reducing AHR and airway inflammation in murine asthma models, their low specificity renders their activity against various other zinc-containing metalloproteins, including numerous non-MMP enzymes and transcription factors, and hence severe side effects leading to their withdrawal from clinical practice. Their low clinical effectivity could also be due to the compensatory induction of other MMPs despite downregulation of the specific targeted MMP.

In addition, various other clinically used drugs are also known to modulate MMP activity either directly or indirectly. Corticosteroids, statins, angiotensin-converting enzyme (ACE) inhibitors, and tetracyclines belong to this category. Dexamethasone, a commonly used corticosteroid, selectively and potently inhibits a variety of MMPs, including collagenases and stromelysins, in addition to TIMPs in human alveolar macrophages [97]. The inhaled corticosteroid (ICS), beclomethasone dipropionate (BDP), attenuated the expression of submucosal MMP-9 and increased that of submucosal TIMP-1, suggesting corticosteroid treatment of asthma to ameliorate angiogenesis [98]. In a murine asthma model, inhaled administration of budesonide significantly reduced the vascularity and the expression levels of HIF-1*α* and VEGF, supporting an anti-angiogenic role for budesonide in the treatment of human asthma [99]. A placebo-controlled intervention study exploring angiogenic modulation upon ICS treatment in patients with asthma revealed a reduction in microvascular angiogenic remodeling in asthmatic airways in terms of a decrease in vessel numbers, VEGF staining, and number of sprouted vessels in airway biopsy specimens [100].

The subject of targeting angiogenesis in asthma is one of considerable debate considering its role in normal physiological functions. During airway and lung growth, they progressively require good blood supply, which is paralleled by the expression of VEGF and its receptors [101]. Therefore, with therapeutic strategies targeting VEGF using anti-VEGF antibody (Avastin) and anti-VEGFR-1 and anti-VEGFR-2 antibodies, such as in cancer therapeutics, arises the possibility of excessive vascular regression that could compromise drug delivery to the target site and set off unwanted side effects [102].

## 8. Future Perspectives

**Figure 1:**
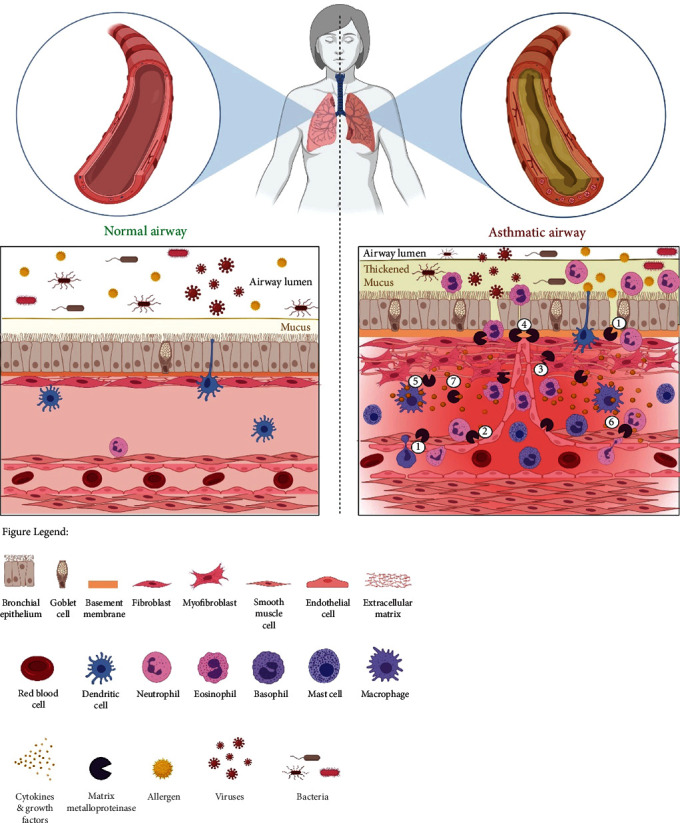
Schematic illustration of the involvement of MMPs in facilitating angiogenesis in asthma. In an asthmatic airway microenvironment, MMPs are involved in (1) the extravasation of inflammatory cells into the airways as well as their egress into the airway lumen across the vascular and airway membranes, (2) the degradation of vascular basement membrane, (3) degradation of ECM components (collagen, proteoglycans, elastin, gelatin, fibrin, fibronectin, aggrecan, and laminin), (4) aiding the endothelial sprouting tip to invade into the surrounding stroma, (5) release of proangiogenic cytokines and growth factors (HIF, VEGF, bFGF, PDGF, and TGF-*β*) from ECM, activated airway structural cells (epithelial, endothelial, fibroblast, and smooth muscle cells), and infiltrating inflammatory cells (eosinophils, neutrophils, basophils, mast cells, and macrophages), (6) regulation of perivascular or smooth muscle cells, and (7) ectodomain shedding of cytokines and their cognate receptors. This complex interplay between the activated cells, MMPs, cytokines, and growth factors directs the development of a vascular network within asthmatic airways. Created with http://BioRender.com/.

Initially thought of as an immunological disorder, asthma is now increasingly appreciated as a disorder affecting the airway wall with aberrant inflammatory and remodeling responses. Asthma is a complex heterogenous disorder with multiple hallmark features, one among which is angiogenesis and vascular remodeling. Angiogenesis appears to facilitate the development of airway edema in the initial stages and further on progress to contribute towards bronchial wall thickening with concomitant reduction in distensibility. MMPs through their actions on ECM degradation and regulation of cell signaling play a role across multiple pathological processes of asthma, including angiogenesis.  Figure 1 illustrates the various pathological roles of MMPs in facilitating angiogenesis in asthma.

Although there are improved research and insights into various aspects of angiogenesis and vascular remodeling in asthma, there are several unanswered questions, answers to which could provide a well-rounded understanding of the implications of MMPs in the pathology of angiogenesis in asthma. In comparison to their healthy counterparts, the various cellular players in asthma secrete increased levels of cytokines, chemokines, growth factors, and angiogenic mediators. Despite the well-known anti-inflammatory activity of corticosteroids, their ability to reverse or reduce airway remodeling continues to remain a subject of controversy. Although high doses of ICS affect certain components of remodeling, increased vascularity, for example [100, 103, 104], they do not uniformly target the various remodeling features. The ability of MMPs in regulating both the inflammatory and remodeling aspects of asthma pathology opens the possibility of completely unexplored avenues of asthma therapy. A detailed understanding of the various members of the MMP family and their contribution towards angiogenesis across the various asthma endotypes and phenotypes is lacking. Depending on the disease endotype, targeting specific MMPs and averting their pathological signaling can be beneficial in that subset of patients. Future studies could also potentially help identify novel angiogenic signaling pathways in asthma, and their regulation by MMPs provides opportunities to categorize the various roles played by MMPs in asthma which could be harnessed for therapeutic intervention. Since miRNAs are increasingly being implicated in angiogenesis and endothelial cell function, the ability of MMPs to modulate specific miRNAs is another interesting avenue for research. The search for a non-invasive therapy capable of reducing or even completely abrogating vascular and other remodeling features is another meaningful approach.

In conclusion, asthma being a multifaceted disease calls for the development of new therapeutic strategies that can target the various remodeling features observed in asthmatic airways. The studies discussed above provide insights into the role of MMPs as a potential target to ameliorate angiogenesis among various other remodeling features. The ability of MMPs to target both inflammatory and remodeling processes makes it an attractive option for therapy. Furthermore, MMP levels and vascularity are also correlated with asthma severity indicating their role in asthma pathogenesis and progression. Several animal studies have further reinforced this data supporting the notion that reducing or reversing vascular remodeling may prove beneficial in treating asthma. Although corticosteroids have potential MMP-mediated anti-angiogenic activities, there is a need for novel strategies targeting MMPs. Targeting MMPs is a novel therapeutic strategy for treating the microvascular changes observed in asthma considering their ability to reduce angiogenesis, inflammatory response, and thereby asthma symptoms. Nevertheless, it is crucial to identify the subset of asthma patients that respond and benefit the most from such an approach.

## Figures and Tables

**Table 1:** MMPs implicated in promoting angiogenesis in asthma.

Name	Substrates	Cellular sources	Functions	References
MMP-9 (gelatinase-B)	Collagens IV, V, VII, X, and XIV, gelatin, pro-MMP-9, pro-MMP-13, elastin, aggrecan, laminin	Bronchial epithelial cells, endothelial cells, fibroblasts, neutrophils, alveolar macrophages, mast cells, eosinophils, dendritic cells, T cells	(i) Triggers angiogenic switch (ii) Cryptic epitope exposure within collagen IV (iii) Cleavage of latent TGF-*β* (iv) Proteolytic activation of IL-8 and inactivation of platelet factor-4 (v) VEGF mobilization from ECM (vi) iNOS activation (vii) Endothelial basement membrane disruption (viii) EC growth & migration (ix) Eosinophil infiltration (x) Pericyte recruitment (xi) Hydrolyze plasminogen to angiostatin	[51, 52, 54, 61, 71–74]

MMP-2 (gelatinase-A)	Collagens I, II, III, IV, V, VII, X, XI, and XIV, gelatin, elastin, fibronectin, aggrecan, CCL7, CXCL12	Fibroblasts, bronchial epithelial cells, smooth muscle cells, endothelial cells, neutrophils, macrophages, T cells	(i) Triggers angiogenic switch (ii) Cryptic epitope exposure within collagen IV (iii) Binding to integrin *α*v*β*3 (iv) VEGF mobilization from ECM (v) Cleavage of latent TGF-*β* (vi) Smooth muscle proliferation (vii) Endothelial basement membrane disruption (viii) Eosinophil infiltration (ix) Hydrolyze plasminogen to angiostatin	[51, 52, 71, 75, 76]

MMP-7 (matrilysin-2)	Collagens II, III, IV, IX, X, and XI, elastin, pro-MMP-1, pro-MMP-7, pro-MMP-8, pro-MMP-9, pro-MMP-13, gelatin, aggrecan, fibronectin, laminin, syndecan-1, E-cadherin	Smooth muscle cells, epithelial cells, macrophages	(i) EC proliferation (ii) Upregulation of endothelial expression of MMP-1 and MMP-2 (iii) CTGF cleavage to release active VEGF^165^ (iv) Endostatin fragment release from ECM (v) Hydrolyze plasminogen to angiostatin	[47, 72, 77–79]

MMP-1 (collagenase-1)	Collagens I, II, III, VII, VIII, and X, aggrecan, gelatin, pro-MMP-2, pro-MMP-9	Fibroblasts, smooth muscle cells, alveolar epithelial cells, endothelial cells, alveolar macrophages	(i) Degradation of interstitial collagen types I-III (ii) Degradation of perlecan in endothelial basement membrane to release bFGF (iii) CTGF cleavage to release active VEGF (iv) Promotes VEGFR2 expression (v) Endothelial invasion capacity (vi) Smooth muscle hyperplasia	[79–81]

MMP-8 (collagenase-2)	Collagens I, II, III, VII, VIII, and X, aggrecan, gelatin	Neutrophils, fibroblasts, endothelial cells	(i) EC proliferation and migration (ii) PECAM-1 expression (iii) Neutrophil clearance	[82]

MMP-14 (MT1-MMP)	Pro-MMP-2, pro-MMP-13, collagens I, II, and III, gelatin, aggrecan, fibronectin, laminin, proteoglycan, CD44, E-cadherin, syndecan-1	Bronchial epithelial cells, endothelial cells, fibroblasts, alveolar epithelial cells, macrophages	(i) Stimulate invasion into collagen (ii) Pericellular collagenolysis (iii) Proteolytic degradation of antiangiogenic factors (such as decorin) (iv) Stimulate VEGF production (v) Induce EC migration	[83–85]

MMP-12 (metalloelastase)	Elastin, collagen IV, laminin	Epithelial cells, alveolar macrophages	(i) Angiostatin production (ii) Hydrolyze plasminogen to angiostatin (iii) Endostatin release	[86, 87]

Abbreviations: TGF: transforming growth factor; iNOS: inducible nitric oxide synthase; EC: endothelial cell; CTGF: connective tissue growth factor; bFGF: basic fibroblast growth factor; PECAM: platelet endothelial cell adhesion molecule; VEGFR: vascular endothelial growth factor receptor.

## Data Availability

No data were used to support this study.
